# Cold-blooded culture? Assessing cultural behaviour in reptiles and its potential conservation implications

**DOI:** 10.1098/rstb.2024.0129

**Published:** 2025-05-01

**Authors:** Anna Wilkinson, Stephan A. Reber, Holly Root-Gutteridge, Angela Dassow, Martin J. Whiting

**Affiliations:** ^1^ School of Natural Sciences, University of Lincoln, Lincoln LN6 7TS, UK; ^2^ Cognitive Science, Lund University, 221 00 Lund, Sweden; ^3^ Department of Biology, Carthage College, Kenosha, Wisconsin 53140, USA; ^4^ School of Natural Sciences, Macquarie University, Sydney, New South Wales 2109, Australia

**Keywords:** social learning, animal culture, conservation, behaviour, non-avian reptile, reptile

## Abstract

It is becoming clear that the cognition of a species plays an important role in successful conservation, with cultural processes being a fundamental part of this. However, in contrast to mammals and birds, very little is known about cultural processes (and the social learning that underlies these) in reptiles. Here, we summarize the current state of knowledge, consider why this information is so limited and assess candidate behaviours observed in the wild, which warrant further investigation through the lens of cultural traditions. We then make suggestions for the fundamental next steps necessary to start to address this issue. This includes future experimental work and also consideration of how existing datasets, such as those capturing animal movement or acoustic activity, can be used to assess cultural questions. In addition, we emphasize the important role that engaging key conservation stakeholders, such as zoos, aquaria and ecotourism providers, could play in furthering our understanding of cultural behaviour in this group and the potential conservation implications of this knowledge. Whether there is cultural behaviour in reptiles and the relationship that this has with conservation remain unclear; however, the findings of this review suggest that these are areas worthy of further research.

This article is part of the theme issue ‘Animal culture: conservation in a changing world’.

## 1. Introduction

Culture was long thought to be unique to humans; however, there is now increasing evidence of cultural phenomena across the animal kingdom ([1], and this special issue). Culture can be defined as the behavioural patterns that are transmitted through socially learned information and shared by members of a group or community [2]. Some authors refer to *traditions* instead of culture. The two are typically considered one and the same, although individual traditions, e.g. migrating to a feeding ground, are sometimes referred to collectively as culture [3]. For further consideration of what constitutes a culture see Whiten & Rutz’s contribution in this special issue [3]. There are now examples of cultural behaviours across the animal kingdom [1], and while these are becoming well understood in some mammal and bird species, there is much less evidence and knowledge in non-avian reptiles (henceforth, reptiles). The role that culture might play in conservation is being increasingly recognized [4,5] and thus it is important to assess the implications of this in reptiles, a group where around 21% of species are threatened with extinction [6].

There are at least 12 263 species of extant reptiles [7], belonging to four different orders (Squamata: lizards, amphisbaenians and snakes; Chelonia*:* turtles, terrapins and tortoises; Rhynchocephalia: tuatara; Crocodylia: crocodiles, gharials, alligators and caimans [8]). Current conservation efforts largely follow traditional routes, including understanding a species’ ecosystem and habitat needs. While extremely valuable, these approaches do not consider crucial factors that determine how animals find and return to resources—the cognitive processes underlying their behaviour [9]. There is therefore a pressing need to improve our understanding of reptile cognition in general and, in this context, social cognition in particular. Behavioural traditions may allow animals to adapt more (or less) readily to change, and the ability to learn from another individual may result in more rapid adaptation to environmental challenges than individual learning or other adaptations.

A fundamental prerequisite for culture is social learning, the process in which individuals gain new information by observing the behaviour of another individual rather than by individual learning [10]. There is very little research investigating social cognition in reptiles. The reason for this is probably twofold. Reptiles have traditionally been considered ‘sluggish and unintelligent creatures’ [11, p. 520] and as such they have largely been ignored in the study of animal cognition. However, when reptiles are tested under appropriate conditions, we now have strong evidence of key cognitive skills in this group, including spatial learning, quantity discrimination and long-term memory [12–16]. Furthermore, reptiles are capable of behavioural flexibility—the ability to solve a novel problem or an existing problem in a novel way [15,17–19]. In addition to the lack of fundamental understanding of their cognitive abilities, reptiles used to be considered primarily solitary and, while some indeed are, we now have a much better understanding of their social structures and their complexity [20–22]. For example, some species live in family groups, which can sometimes be multi-generational [20,23]. The parental unit in these groups is largely monogamous. In one species, the sleepy lizard (*Tiliqua rugosa*), males and females pair for life but only come together for a few months during the breeding season [24]. Some species exhibit complex alternate social and reproductive tactics while others exhibit the 'dear enemy' phenomenon and/or individual recognition [25–29]. The social networks and relationships of squamates and other reptiles highlight the potential importance of social information in their decision-making processes [21,30,31], something that is likely to impact the success of conservation interventions. Taking all of the above into account, there is good reason to believe that social learning is more widespread in reptiles than we might currently appreciate. This paper will review this evidence and consider the conservation implications of these findings together with key next steps for future work.

## 2. Current knowledge

### (a) Using social information: gaze following

The ability to align gaze direction with that of another individual is considered adaptive as it is likely to alert the observer to the presence of important stimuli in the environment, such as food or predators. There are two different modes of gaze following: gaze following into distant space and geometric gaze following. The former requires an animal to reorient its gaze to where another individual is looking. This is likely to be controlled by a simple orienting response accompanied by learning. There is evidence of this ability in Chelonia (red-footed tortoises, *Chelonoidis carbonaria* [32,33]), Squamata (bearded dragons, *Pogona vitticeps* [34]; leopard geckos, *Eublepharis macularius* [35]) and Crocodylia (American alligators, *Alligator mississippiensis* [36]). In contrast, geometric gaze following is considered cognitively complex as it requires an animal to assess the difference in the visual perception between themselves and the cue giver. This is generally assessed by the cue giver looking behind a barrier, thus, the observer needs to reposition itself to see what the cue giver is looking at [34,36]. While bearded dragons and alligators have failed to demonstrate this using standard paradigms, there is very recent evidence that red-footed tortoises are able to follow the gaze of a conspecific around a barrier [33], suggesting that they can assess the difference in perception between themselves and another individual. In addition, there is some indirect evidence of this ability in alligators [32]. When the gaze of demonstrators was directed toward a target above the animal’s head, instead of looking up, the observers turned their heads to look behind themselves, potentially aligning their field of vision with that of the demonstrator, and they did so significantly more often than in control conditions. Taken together, this work demonstrates cases where reptiles use social information to alert them to changes in the environment—important pre-conditions for social learning. Furthermore, at least one species is able to perceive the differences between their perspective and that of another individual, suggesting that they are capable of sophisticated social cognition.

### (b) Social learning

Social learning is considered adaptive as it can provide a shortcut to solving a challenge without the costly process of trial-and-error learning [37]. There is evidence that chelonians can learn from observing a conspecific (e.g. [32,38]). Red-footed tortoises can learn a detour task, which they cannot otherwise solve, by observing a conspecific make the detour. The social learning mechanisms that control this behaviour remain unclear; however, tests revealed that the tortoises learned general principles about how to solve the task rather than a specific route [32,39].

Conspecific social learning has been tested for in a small number of lizard species, with differing ecology and social behaviour [40–43]. Bearded dragons (*Pogona vitticeps*) are mostly solitary, with a polygynandrous mating system. They use both colour and motion-based visual signalling [44,45]. Although they do not aggregate, local densities can be high, such that individuals on elevated perches are within view of others in their neighbourhood. Thus, there is an ecological context ripe for social learning. Social learning was tested for in a captive colony of bearded dragons. Bearded dragons in a ‘social learning’ treatment observed a demonstrator open a sliding door while the control group observed the door open with another lizard present but inactive and uninvolved with the opening of the door. All eight animals in the social learning treatment opened the sliding door while none of the control group did. Furthermore, all eight did so in the same direction as the demonstrator, confirming that they used imitation to achieve this goal [41]. Social learning was also tested for in two Australian skink species: the family-living tree skink (*Egernia striolata*) and the mostly solitary eastern water skink (*Eulamprus quoyii*) [42,43]. Testing in both species involved learning to first displace a lid from a food dish, followed by an associative learning task (colour discrimination) in which the social learning treatment observed a demonstrator remove a lid from only the correct (food reward) dish. In both species, the social learning treatment group made fewer mistakes and reached the learning criterion faster. Only adult females were tested in the family-living tree skink, while only males of two age cohorts (subadult, adult) were tested in water skinks. Interestingly, in the water skinks, young subadult males used social information to learn faster while adult males did not. Adult males compete; thus, social feedback may constrain social learning. Although water skinks do not aggregate, like bearded dragons, they can occur at high densities. Their close proximity to one another gives them easy access to social information from other individuals in their neighbourhood.

Both conspecific and heterospecific social learning have been demonstrated in the invasive Italian wall lizard (*Podarcis sicula*) [46]. Wall lizards were able to learn a foraging task faster by using social information and it made no difference if the demonstrator was a conspecific or a heterospecific (*Podarcis bocagei*). Compared with individual learners, social learners made fewer mistakes. Overall, a relatively low proportion of individuals were able to solve the task, although a greater proportion were social learners (social learners: 38%, 8/21; individual learners: 13%, 2/16) [46]. Furthermore, this species has been shown to use social information even when it conflicts with individually learned information [47], suggesting that it is likely to have a strong impact on subsequent behaviour. Furthermore, insular lizards (*Podarcis lilfordi*) that live in high population densities are more likely to approach food where a conspecific is foraging, both when real conspecifics are used and when a model is used [40]. Given that wall lizards readily use social information and, as an invasive species with range expansion, are more bold, exploratory and neophilic than congeneric native species [48], they may be a particularly good model system for investigating culture. The role that culture may play in the success of invasive species is a crucial question to investigate in future work.

There is evidence that juvenile painted turtles (*Chrysemys picta*) likely navigate using ultraviolet information from the sloughed skin and faeces of adults [49]. However, this ability appears to be transitory as individuals over 4 years of age are unable to do this, suggesting a critical period for learning from social cues in this species [49,50]. As mentioned above, Noble *et al*. [42] also found evidence of age-dependent social learning in lizards, with subadult male skinks (*Eulamprus quoyii*) benefitting from observing a demonstrator while adult males did not. Should these findings be more general, then this has crucial implications for the spread of cultural information in reptiles. Furthermore, if animals are only able to use these social cues in a critical period during their development, this would suggest that changes to an environment, such as a change in food sources or displacement, are likely to impact adults more substantially than juveniles. At the same time, knowledgeable individuals are essential for the success of younger individuals—thus this is likely to have substantial conservation implications, particularly in reintroduction success.

In terms of development, reptile embryos experience a wider range of temperatures compared with mammals and birds. These can impact various aspects of phenotype relevant to their social cognition. Incubation environment has been shown to impact the development of behavioural traits [51,52], neuronal development [53] and operant conditioning [51,54,55]. The rate of social learning (but not gaze following) in adult bearded dragons also differs depending on their incubation environment [34]. Animals were incubated at two different temperatures, both within the normal range of the species, and those that were incubated at the cooler temperature were both quicker at learning a social learning task and faster at completing that task than animals incubated at a warmer temperature. Thus, small differences in the early developmental environment can have a long-lasting impact on fundamental aspects of cognition and subsequent fitness in reptiles. This has important implications in the context of both conservation and cultural transmission. It may be that the likelihood of cultural transmission occurring in reptiles depends not only on the socio-cognitive abilities of the species in question but also on an individual’s developmental environment as an embryo. Furthermore, it is possible that the differences in cognition that result from differing incubation environments may act as a behavioural buffer, potentially allowing oviparous reptiles to more readily adapt to environmental change.

## 3. Communication and culture?

Communication is one of the main domains where cultural transmission in reptiles may occur. Reptiles are highly diverse in their modes of communication; here, we focus on visual, chemical and acoustic communication as these are more readily observed and frequently used in a social context and thus more likely to be amenable to cultural transmission.

### (a) Visual communication

Lizards are well known for a wide range of motion-based signals, which include dewlap extensions, head bobs, tail waves and flicks, arm waving, lateral or ventral displays and push-ups [56–58]. Furthermore, these displays may occur in combination, and in some species they include colour signals [59,60]. Visual signals are thought to play a key role in species recognition [61] and can act as signals of social dominance or fighting ability [62], and potentially as indicators of quality, although this is less well understood. Although more unusual in snakes, there is evidence that displays may also be a mark of the quality of a competitor [63]. In lizards, there appears to be variation in display behaviour between populations of the same species [64–66] and even evidence of different behaviours used within a display across different populations [66]. The role that learning plays within this remains unclear.

With respect to species recognition, sagebrush lizards (*Sceloporus graciosus*) appear to be sensitive to the ‘grammatical’ structure of their species’ head-bobbing behaviour. In an experiment with robotic lizards (capable of head bobbing), subjects showed more aggression to the robot if it produced bobbing sequences violating their species’ convention [67]. Still, lizard head bobbing seems to be idiosyncratic and less flexible. If there is a learned component, which could also be culturally transmitted, it might occur in a temporally limited sensitive phase of an animal’s ontogeny. Given that the bearded dragon is a head-bobbing member of the Iguania and can socially learn by imitation [41], this behaviour could be a worthwhile avenue for future studies on reptile culture.

### (b) Chemical communication

Chemical communication plays an important role in mediating social behaviour, particularly in squamates [68–70], and potentially social learning, and has been shown to facilitate group cohesion [22,68,71] and site selection [72] in reptiles. Chemical communication is widespread among the squamates. In a social context, chemical cues may be used for species recognition, mate assessment, rival recognition, individual recognition and kin recognition [20,27,69,73–79]. It has even been posited that there is evidence of chemical self-recognition in snakes [80,81], although the mechanisms underlying this are still debated. Taken together, this shows that chemical social information is highly relevant to this group and suggests that the area is ripe for future research relating this to social cognition.

### (c) Acoustic communication

While sounds can be produced in many ways, vocal production in amniotes requires a sound source, which is the vibrating tissues of the larynx (in reptiles and mammals) or the syrinx (birds). All reptile species possess a larynx and might therefore be capable of producing at least a limited repertoire of vocalizations [82]. Known reptile calls include roars, squeaks, clicks, and harsh, deep croaks [83–86]. Vocal communication might have arisen, and potentially re-arisen independently multiple times, in lepidosaurs, chelonians and crocodilians [82]. Despite this, most reptiles have traditionally been considered non-vocal. Our knowledge of acoustic communication varies widely across taxa, from the very limited knowledge of the tuatara (*Sphenodon punctatus*) to the comparatively well studied crocodilians. Sound can communicate a wide variety of information about the caller, including sex, individual identity, age and location, as well as the behavioural context, e.g. reproductive and/or territorial [87]. While some call features are defined by the caller’s anatomy, others may be under at least partial control of the caller. Communication is thus an important area when considering cultural processes in this group.

Many crocodilians produce vocalizations during social interactions, such as maintaining crèche cohesion, courtship displays and territory defence [85,88]. Hatching, distress and disturbance calls are used by young crocodilians to elicit maternal protection [89,90]. All crocodilian species start vocalizing in the egg to synchronize hatching and to recruit parental protection [91], but there is currently no evidence of learned components to these or other juvenile calls, which precludes discussions on cultural transmission. However, adult advertisement calls are much more diverse, and their perception is barely studied. For instance, the bellowing displays of American alligators carry reliable cues of caller size and sex [92], and they are highly individually distinct [93]. These calls might play a role in recruiting receivers to previously unexplored areas and hence in cultural transmission. They could also be a useful tool for researchers to track the whereabouts of individual animals.

Similar research in other reptile taxa is constrained by a lack of basic natural history knowledge. Our understanding of chelonian vocalizations is still extremely limited [94–96]; at least seven species of turtles are capable of vocalizing by the time they hatch [97–103]. The functions of these calls remain largely unexplored, although in at least one species, the Arrau turtle *Podocnemis expansa*, they appear to play a role in eliciting parental care or in synchronizing hatching [99], something that has been proposed in multiple reptilian species [91,96–98,104]. Chelonian vocal repertoires are thought to be generally limited to a few call types, although in one species, the eastern long-necked turtle *Chelonia longicollis*, the repertoire consists of 17 categories of sounds [96]. It may be that this species is a taxonomic anomaly; however, it is also possible that the vocal repertoires of chelonians are larger than we realize, and this is an artefact of the under-sampling of this group. Exploring a wider range of species of Chelonia could offer better insight into this matter.

Among squamates, geckos are famous for being vocal [82,105]. Given that all other groups within Squamata have very few vocalizing species, it appears that there is little selection for vocalization more broadly. Snake hearing organs are not sensitive to the sounds that snakes produce; therefore, vocal intra-species communication is at best unlikely in snakes [106]. The majority of the other squamates can hear airborne sounds and produce acoustic signals. Many lizards squeak or hiss, but tonal vocalizations are rare [82,84]. Nevertheless, vocal communication has evolved independently in certain lineages. For example, the genus *Liolaemus* (iguanian lizards) contains over 220 species and only a single one, the weeping lizard, *Liolaemus chiliensis,* is known to vocalize [107]. When attacked by a predator, weeping lizards produce distress calls with a clear harmonic structure. The call can deter the predator or attract a secondary predator, and close-by conspecifics react to it by freezing to avoid detection. These vocalizations also contain nonlinear phenomena such as chaos (perceived as ‘harshness’) and are both lower-pitched (fundamental and dominant frequency) and longer in larger individuals [107]. The acoustic parameters vary with the geographical distribution, and weeping lizards show distinctive responses to them [108]. This makes this species a good candidate for a cultural behaviour. While the evolution of this communication system did not require cultural transmission, as direct fitness benefits arise from the behaviours of producer and receiver, it has been hypothesized that *L. chiliensis* evolved acoustic communication because it lives in bushes with limited visibility, which is an uncharacteristic habitat for the genus [107]. However, selection can only work with pre-existing traits, and it is rather improbable that a random mutation just happened to occur in the one species in which it would be most beneficial, particularly as other species in more open habitats would still profit from such a communication system. So, closely related species might have a similar potential to acquire vocalizations. These systems do not necessarily require cultural transmission to arise, but given that they are social and novel behaviours, there is potential that culture could play a role in shaping them.

We are not aware of any evidence for vocal production learning in reptiles; however, some populations might use certain elements of their call repertoire more frequently or sequences of signals might be differently composed between populations. Tokay geckos (*Gekko gecko*) increased the duration of their call syllables in noise, producing 7% longer GECK syllables and 37% longer O syllables [109]. They also increased the number of loud GECK-O calls more than the soft crackles in noise. This shows that Tokay calling behaviour is not static and is adapted to specific contexts, demonstrating flexible vocal production is possible in reptiles and thus not limited to certain mammals and birds [109]. However, it has not yet been established if the Tokay gecko is a unique case or representative of other squamates.

Thus far, we have established that reptiles can produce and use a range of vocal and non-vocal acoustic signals. Following Lin *et al*. [110], we suggest that these signals may be used as mediators of complex social behaviours. Exploring how these signals may vary across populations could inform us about cultural behaviours in this group.

## 4. Future avenues for assessing cultural behaviour in reptiles

Below we have compiled a candidate list of reptiles for assessing cultural behaviour, and consider the next steps for assessing this in these species and beyond. We include suggestions for new research directions and ideas for using existing datasets, such as those capturing animal movement or acoustic activity, to investigate cultural questions.

### (a) Social learning in the wild: opportunity and importance

Food resources can vary in space and time. For many species, searching for food is constrained by energetic costs and a greater exposure to predation risk. Thus, using social information is a means of reducing both these constraints, and foraging behaviours are an excellent candidate for observing cultural behaviour. For example, Augrabies flat lizards (*Platysaurus broadleyi*) feed on both insects (particularly flying insects such as black flies, *Simulium* sp.) and small Namaqua figs (*Ficus cordata*) [111,112]. In the case of black flies, they occur in fast-flowing sections of rivers, where large plumes of male flies gather to wait for emerging females. This clumped food source is a boon for the lizard, and individuals travel from refuges and territories away from the river to these feeding areas along the same path, which is shared by multiple individuals. Interestingly, this species is a sit-and-wait forager that travels between food patches. Namaqua fig trees fruit asynchronously and are thus unpredictable. However, when discovered by lizards, the animals will return repeatedly until the resource is depleted. Lizards are thought to use social information from foraging birds to know when food is available [113], although it is also possible that they use information from other conspecifics travelling a particular route. These lizard highways are arguably a tradition that could be socially learned.

Saltwater crocodiles (*Crocodilus porosus*) temporarily aggregate at a certain concrete causeway when king tides coincide with the spawning migration of flathead grey mullet (*Mugil cephalus*). The higher water levels enable the fish to cross the man-made obstacle, but as the water is shallow, they are easy prey for resident crocodiles [114]. Saltwater crocodiles are highly territorial and such aggregations are relatively uncommon [115]. In addition, they seem to travel over large distances (over 45 km for at least one tracked animal) at an appropriate time (prior to the king tide) to access the food source [114]. So, how do the aggregating crocodiles know where this spot is and when to be there? It is theoretically possible that they all individually discovered this feeding spot and coincidentally encountered it at the right time and/or they might use cues that allow them to predict tides. However, they might also have observed knowledgeable individuals travel past their territories and followed them to potentially exploit a new food source, which could lead to a spread of this behaviour through local populations.

Many examples of culture in mammals and birds relate to novel foraging techniques [1]; therefore this is an important avenue for exploration in reptiles. One potential candidate for this is an interesting and unusual foraging behaviour observed in saltwater crocodiles, the ‘cross posture’ or ‘cross foraging posture’, which involves the animal extending its forearms perpendicularly from its body with palms facing outwards and digits facing upwards, above the water, while its mouth is open with the bottom jaw below the water and the upper jaw above the water. This behaviour is very distinct and has only been reported in adults from a few locations in the Australian Northern Territory and Western Australia and from juveniles in the Daintree River of Queensland [116,117]. It is commonly observed in one population at Cahill’s Crossing in Kakadu National Park in the Northern Territory. It is unclear what mechanism is controlling this behaviour but, given the patchy sightings of it, it could represent a local tradition and warrants further investigation as a potentially socially learnt behaviour.

Sea turtles represent another opportunity for studying traditions because they are famous for migrating large distances (thousands of kilometres, up to almost 10 000 km in some cases) to breeding or feeding grounds (e.g. seagrass beds). A multi-year satellite tracking study of 10 green (*Chelonia mydas*) and 10 loggerhead (*Caretta caretta*) turtles migrating to feeding grounds following nesting on the island of Cyprus showed that they very closely match the same route each time. In a review of turtle migration studies, the key finding was that all species and populations studied showed very strong site fidelity to feeding grounds which they returned to repeatedly [118]. Surprisingly, migrating individuals will travel through areas used by other turtles to reach their own, previously visited site, foregoing potential foraging opportunities. Sea turtles use ‘magnetic maps’ to navigate to distant breeding and feeding areas and are thought to also use local cues once they get closer to their final goal. And as they age, they may use more information gained from experience, while navigating back to a resource [119,120]. Green sea turtles spend the first years of their lives locally, not too distant from their natal beaches, before undertaking long migratory movements to seagrass beds many hundreds or thousands of kilometres away. How do they find their way to distant areas they have never previously visited? There may be a range of mechanisms involved, including using social information that may help generate this tradition. Recent work has suggested that the frequency of conspecific social interactions is much higher than previously believed, making it more likely that they may pay attention to social information from other turtles [121–123]. Sea turtles that are born on the same beach and migrate to the same feeding grounds would make excellent candidates for assessing cultural behaviour. Sea turtles may also produce sufficient vocalizations to warrant investigation of social vocal learning and cultural variation within this. Thus, they represent a strong opportunity to investigate cultural processes in reptiles, as they have populations that are genetically distinct and geographically separate. Also, they are long-lived [124].

Reptile species that move between prey or food patches, or even between nesting areas or other suitable microhabitats, represent opportunities for social learning and generating traditions. These observations from phylogenetically distinct species of reptiles suggest that there is a strong potential cultural phenomenon in this group. This, in combination with the data on social learning in other species of reptiles, suggests that cultural phenomena may be relatively common in this group. However, this is a hypothesis, which it is now essential to test.

## 5. Crucial next steps to investigate reptile culture

In order to develop efficient, predictive and accurate conservation tools, it is important to account for the cognitive processes impacting animal decision making and the role that culture plays within this. It is therefore essential to increase our understanding of the role that cultural processes may play in reptile behaviour. Below, we present the key next steps necessary to do this.

### (a) Controlled captive studies

Controlled captive studies with species of particular interest is an essential next step for the field. There is currently no evidence of social learning in Crocodylia. Considering that they demonstrate complex social behaviour and are highly opportunistic hunters, they are excellent candidates for looking at the potential for culture. In addition, sea turtles are of particular interest given their migratory patterns, longevity and conservation status. Thus, replicating some of the studies that have demonstrated evidence of social learning in reptiles already is an essential next step in these groups.

The work presented above clearly shows that individuals of some species of reptiles adapt their behaviour in the presence of another individual and are able to learn from observing the behaviour of a conspecific. A key next step in relating this to conservation is to manipulate the knowledge state of both the demonstrating individual and the observer. Thus, it is essential to replicate with reptiles some of the classic studies used to assess this in mammals. If we can understand factors that make social learning more or less likely, then we can start to understand how this will influence group behaviour in relation to conservation. Furthermore, it will allow us to start to assess the impact that losing specific individuals from a population may have on subsequent success.

Studies of vocalization represent an opportunity to better understand the link between culture and conservation. For example, comparisons of vocal behaviour in captive settings pre- and post-introduction of new individuals could illuminate flexibility in vocal production and repertoire use. Whether new calls are adopted or existing repertoires are used differently is an interesting question relating to learning and cognition. If these differences do exist, then they may have a marked influence on the behaviour of different populations.

### (b) Studies in the wild

It is also important to experimentally assess social learning and transmission of behaviour in wild populations. One method could be to create controlled new food patches and monitor individual movements in a social network. These patches could be long-term or could be ephemeral, and predictable or unpredictable. This would allow assessment of the factors that may encourage behavioural traditions to occur and situations in which they are more likely. Bioacoustics could be used to track the whereabouts of individual crocodilians in a larger area. The basic software for passive acoustic monitoring of American alligator bellows has already been created [93] and at least suggested for African dwarf crocodiles (*Osteolaemus tetraspis*) [125]. Passive acoustic monitoring of this type has been widely used for a wide range of other vocal taxa, and training detectors to recognize reptile calls as part of acoustic indices could benefit detection and survey efforts. When complemented with other monitoring methods, it offers an additional tool for long-term monitoring of populations. It can also be used to track and explore differences in the vocal behaviour of distinct populations, mapping changes over time or with geography, and can be tied to population flow, providing crucial measures for monitoring the need for interventions or the success of conservation efforts.

In addition, it is fundamentally important to assess the spread and persistence of socially transmitted behaviours in the wild. By introducing alternative foraging techniques within populations, we can assess the spread and persistence of the behaviours within a group and between groups [126]. This, alongside measures of social interactions, allows us to assess the factors that may impact the diffusion of the introduced behaviours [126].

## 6. Use of existing datasets to ask cultural questions

### (a) Bioacoustic data

The acoustic soundscape has been closely monitored in a number of species, most notably cetaceans [127,128] and numerous terrestrial animals [129], and such data can be harnessed for assessing the presence and distribution of reptiles such as sea turtles—a group only recently gaining attention for vocal communication [130–133]. Using this wealth of pre-existing data from broadscale bioacoustics monitoring, we could assess the presence and absence of different species, the existing vocal repertoire and potentially the development of new calls over time. Furthermore, by using data collected in different regions, geographical variation can be identified and comparisons made across sites. These data could also potentially be used to make phylogenetic comparisons of vocal production, examining whether call evolution has converged on the same repertoire for the same contexts.

### (b) Sea turtle movement data

The vast amount of location and movement data available from tracking sea turtles could be used to assess social interactions and, potentially, cultural behaviour. Recent research has revealed that spatial network analyses can identify both structural and functional connections between six sea turtle species across multiple sites [134]. By harnessing these data to assess the frequency of interactions and foraging, movement data could additionally provide a wealth of information on social cognition, behaviour and, potentially, culture. A first step could be to use these data to create social networks that investigate whether turtles are more likely to spend time with specific individuals rather than in specific places as these data are currently used.

### (c) Engaging stakeholders

One of the greatest challenges of working with endangered species is accessing the numbers necessary to ask the fundamental questions highlighted above. One possible solution is to solicit help from conservation programmes already working with the species of interest. Thus, a key next step is to create accessible, robust and standardized protocols to assess social learning across reptile species. This would allow us to collect data across a wide range of species where no data exist currently and would both widen and deepen our understanding of social learning in this group. In addition, where animals are released (or re-released) and tracked after the tests, we could relate their performance on the tasks to post-release measures of success. Thus, by engaging key conservation stakeholders, such as zoos, aquaria, rescue centres and ecotourism providers with data collection, we would hope not only to fill the substantial scientific knowledge gaps but also to promote the integration of cultural research and knowledge into existing conservation programmes.

## 7. Conclusion

There is a paucity of knowledge of cultural processes, and the social learning that underlies them, in reptiles. However, the research that is available demonstrates that diverse reptile species use social learning. We suggest that social learning need not be restricted to visual cues. For example, reptiles show evidence of complex vocal behaviour and many species rely heavily on chemical cues as sources of information. Furthermore, we have provided observations of situations that could be examples of culturally transmitted behaviour in the wild. Investigating the presence of cultural transmission in reptiles will require further research under controlled captive conditions, research in the wild and the use of existing datasets to assess cultural questions. Thus, whether there is cultural behaviour in reptiles and the relation that this has to conservation remain open questions, but questions that are very much worth exploring.

## Data Availability

This article has no additional data.
